# Calculating an estimate of tissue integrated activity in ^18^F-FDG PET imaging using one SUV value

**DOI:** 10.1186/2191-219X-3-26

**Published:** 2013-04-05

**Authors:** Eric Laffon, Manuel Bardiès, Jacques Barbet, Roger Marthan

**Affiliations:** 1 Université de Bordeaux, Centre de Recherche Cardio-Thoracique, INSERM U-1045, Bordeaux, 33076, France; 2 INSERM, U-1045, Centre de Recherche Cardio-Thoracique, Bordeaux, 33076, France; 3 CHU de Bordeaux, Services de Médecine Nucléaire et Explorations Fonctionnelles Respiratoires, Bordeaux, 33000, France; 4 INSERM, U-892, Centre de Recherche en Cancérologie de Nantes-Angers, Nantes, 44000, France; 5 Université de Nantes, Nantes, 44000, France; 6 CNRS, UMR 6299, Nantes, 44000, France

**Keywords:** ^18^F-FDG dosimetry, Integrated activity, Kinetic modelling, SUV

## Abstract

**Background:**

A kinetic model analysis was recently proposed to estimate the 18F-fluorodeoxyglucose (^18^F-FDG) integrated activity in an arbitrary tissue that uses tracer uptake and release rate constants. The aim of the current theoretical paper was to estimate ^18^F-FDG integrated activity using one standardized uptake value (SUV).

**Methods:**

A further kinetic model analysis allowed us to derive an analytical solution for integrated activity determination, involving both irreversible and reversible trapping. It only uses SUV, which is uncorrected for ^18^F physical decay (SUV_uncorr_, in g.mL^−1^) and is assessed about its peak value. Measurement uncertainty of the estimate was also assessed.

**Results:**

In a tissue (volume *V*, in mL) that irreversibly traps ^18^F-FDG, the total number of disintegrations can be estimated as: *Ã*_C_ = 162 * 10^5^ * SUV_uncorr_ * *V* * *ID* / *W* (*ID*, injected dose, in MBq; *W*, patient’s weight, in kg), where SUV_uncorr_ is a mean over *V* and is assessed between 55 and 110 min after tracer injection. The relative uncertainty ranges between 18% and 30% (the higher the uptake, the lower the uncertainty). Comparison with the previous Zanotti-Fregonara’s model applied to foetus showed less than 16% difference. Furthermore, calculated integrated activity estimates were found in good agreement with Mejia’s results for healthy brain, lung and liver that show various degrees of tracer trapping reversibility and various fractions of free tracer in blood and interstitial volume.

**Conclusion:**

Estimation of integrated activity in an arbitrary tissue using one SUV value is possible, with measurement uncertainty related to required assumptions. A formula allows quick estimation that does not underestimate integrated activity so that it could be helpful in circumstances such as accidental exposure, or for epidemiologic purposes such as in patients having undergone several examinations.

## Background

^18^F-fluorodeoxyglucose positron emission tomography (^18^F-FDG PET) imaging has become indispensible for managing many diseases, either malignant or benign [1,2]. However, in all nuclear medicine procedures, it is important to assess the absorbed dose deposited from internally distributed radionuclides. This assessment requires combination of integrated activity in source regions and of the so-called *S* values that relate mean absorbed dose in an arbitrary region to integrated activity in source regions [3,4]. Since the level of irradiation induced by diagnostic examinations remains well below the threshold of appearance of deterministic effects, a degree of simplification can be accepted for absorbed dose determination. Tables of *S* values derived from anthropomorphic mathematical phantoms are given in MIRD pamphlets for various radionuclides and organs. Average integrated activity, i.e. the total number of disintegrations that occur from the time of tracer administration (zero) to (theoretically) infinity, or the mean residence time (ratio of integrated activity to injected activity), can be derived from healthy volunteer studies, or from a number of examinations in patients [5]. ‘Model-based’ dosimetric approaches are usually considered as sufficient to deduce a first order estimate of irradiation induced by the nuclear medicine procedure [6]. However, even in current clinical ^18^F-FDG PET imaging, getting a better estimate (i.e. more patient-specific) of the absorbed dose may be relevant, although the only available parameter for ^18^F-FDG uptake is semi-quantitative, i.e. the standardized uptake value (SUV) index. As an example, a first estimation has been made *a posteriori* by Zanotti-Fregonara et al. (Z-F) for an ^18^F-FDG examination accidentally performed during pregnancy [7,8]. It could also be helpful for epidemiologic purpose such as in patients having undergone numerous examinations.

A kinetic model analysis was recently proposed to calculate the integrated activity in an arbitrary tissue for ^18^F-FDG PET imaging, and its efficacy was demonstrated in the brain [9]. That study used ^18^F-FDG uptake and release rate constants for grey matter and white matter, which were calculated from literature data involving dynamic acquisitions, i.e. involving several measurements [10]. In comparison, the aim of the present theoretical work was to investigate whether an estimate of ^18^F-FDG integrated activity in an arbitrary tissue can be computed by only using SUV obtained from a single static acquisition. For this, an analytical solution derived from a kinetic model analysis was established, involving a population-based input function. This analytical solution allows determination of integrated activity that only uses SUV uncorrected for ^18^F physical decay (SUV_uncorr_) and assessed about its peak. A formula was derived that was compared to that of Z-F and its results for foetus, assuming irreversible trapping [7,8]. Furthermore, estimates for healthy brain, lung and liver that show various degrees of tracer trapping reversibility and various fractions of free tracer in blood and interstitial volume, were calculated from this analytical solution and literature data, and were compared to results published by Mejia et al. [11]. This work also assesses the measurement uncertainty of the integrated activity estimation that is related to required assumptions.

## Methods

### Kinetic model analysis

Let us define the SUV at time *t*, normalized to body weight, and *corrected for*^*18*^*F physical decay*, i.e. SUV_corr_(*t*) (g.mL^−1^) [12]:

(1) $${\mathrm{SUV}}_{\mathrm{corr}}\left(t\right)=A_{\mathrm{Tot}}\left(t\right)\;W/\mathrm{ID}$$

where *A*_Tot_(*t*) is the whole ^18^F-FDG activity per tissue unit volume at time *t* (kBq.mL^−1^), which is *corrected for*^*18*^*F physical decay* (and includes trapped tracer and free tracer), *W* is the patient’s weight (kg), and *ID* is the injected dose (MBq).

First, the dosimetry purpose of this work requires calculation of the area under the curve (AUC) of the tissue activity changes with time, i.e. the AUC of the so-called tissue time activity curve (TAC). For this, the results of a previous study are summarized below [9]. A two-compartment model analysis was previously developed to assess radiotracer uptake in tissues, assuming constant uptake and release rates, *K* (min^−1^) and *k*_R_ (min^-1^), respectively (in comparison with the three-compartment model of Sokoloff et al. [13], *K* is (*k*_1_*k*_3_) / (*k*_2_ + *k*_3_) and *k*_R_ is (*k*_2_*k*_4_) / (*k*_2_ + k_3_)). The rate of trapped radiotracer change per tissue unit volume at steady state, i.e. d*C*_Trap_ / d*t* (mL^−1^ min^−1^), is described by the following differential equation:

(2) $$dC_{\mathrm{Trap}}\left(t\right)/dt=KC_{p}\left(t\right)-k_{R}C_{\mathrm{Trap}}\left(t\right)-\lambda C_{\mathrm{Trap}}\left(t\right)$$

where *C*_p_(*t*) is the number of tracer molecules per plasma unit volume at time *t* (mL^−1^), and λ is the ^18^F physical decay constant (min^−1^). Equation 2 yields the TAC of trapped ^18^F-FDG per tissue unit volume at time *t*, i.e. *A*_Trap_(*t*):

(3) ATrapt=λCTrapt=K∑i=13λCi(e−λ+kRt−e−αit)/αi−λ−kR

where *C*_i_ and α_i_ are the coefficients of the ^18^F-FDG input function (IF), which is usually assumed to be a three-exponential curve [14,15]. Such a shape for the IF allows a simple analytic integration of *A*_Trap_(*t*) from zero to infinity, providing integrated activity for trapped ^18^F-FDG per tissue unit volume (mL^−1^). Furthermore, adding the part of free ^18^F-FDG in blood and reversible compartment, i.e. *F* (no unit), and hence extending the initial two-compartment model to a three-compartment model, provides total integrated (cumulated) activity for ^18^F-FDG, i.e. the total number of disintegrations *Ã*_C_ (no unit) occurring in a tissue volume (*V*/mL) [9]:

(4) $${\tilde{A}}_{C}=\;V*\left[\right)F+K/\;\left(\lambda +k_{R}\right)\;\left(\right]*\sum_{i=1}^{3}\left(\;{\mathrm{\lambda C}}_{i}/\alpha_{i}\right)\;$$

where ‘Σ(λC_i_/α_i_)’ is the input function AUC of the tracer (AUC_IF_; in mL^−1^).

Second, because in the present framework the only available parameter for ^18^F-FDG uptake is the SUV, it is then necessary to focus on the ratio *K* / (λ + *k*_R_) (i.e. uptake / (decay + release)) in the right hand side of Equation 4 and to find a further relationship between the parameters. Therefore, let us consider the following equation that temporarily put aside *F*:

(5) $${\tilde{A}}_{C}≅\;V*\;\left[K/\;\left(\lambda +k_{R}\right)\right]\;*{\mathrm{AUC}}_{\mathrm{IF}}$$

and let us write that at the trapped tracer peak, i.e. when d*C*_Trap_ / d*t* = 0, Equation 2 yields:

(6) KCptpeak=λ+kRCTraptpeak

As a result, the combination of Equations 1, 5 and 6 provides the following expression for ^18^F-FDG integrated activity in a tissue:

(7) $$\begin{array}{l} {\tilde{A}}_{C}≅\;{\mathrm{SUV}}_{\mathrm{uncorr}}\left(t_{\mathrm{peak}}\right)\;*\;\left[\;V\;*\;\mathrm{ID}/W\;\right]\;*\\ \left[\;{\mathrm{AUC}}_{\mathrm{IF}}/{\mathrm{\lambda C}}_{p}\left(t_{\mathrm{peak}}\right)\;\right] \end{array}$$

where SUV_uncorr_(*t*) is SUV that is *not corrected for*^*18*^*F physical decay*. It should be noted that the use of SUV_uncorr_(*t*_peak_) in Equation 7, i.e. the use of *A*_Tot_(*t*_peak_) instead of *A*_Trap_(*t*_peak_), involves the activity of both trapped and free tracer in blood and reversible compartment, the latter being related to *F* that was temporarily put aside in Equation 5.

Third, deriving a formula from Equation 7 requires that the second ratio appearing in the right-hand-side of Equation 7 be calculated. In this connection, this ratio can be expressed by means of a normalized input function for injected dose and initial distribution volume, i.e. [AUC_NIF_ / λ*C*_pN_(*t*)], as proposed by Vriens et al. from a patient population [15]. Thus,

(8) $${\tilde{A}}_{C}≅{\mathrm{SUV}}_{\mathrm{uncorr}}\left(t_{\mathrm{peak}}\right)*\;\left[V*\mathrm{ID}/W\;\right]\;*\;\;\left[\;{\mathrm{AUC}}_{\mathrm{NIF}}/{\mathrm{\lambda C}}_{\mathrm{pN}}\left(t_{\mathrm{peak}}\right)\;\right]\;$$

Alternatively, Equation 8 can be expressed by using Equation 1 as

(9) $${\tilde{A}}_{C}≅A_{\mathrm{Tot.uncorr}}\left(t_{\mathrm{peak}}\right)*V\;*\;\;\left[\;{\mathrm{AUC}}_{\mathrm{NIF}}/{\mathrm{\lambda C}}_{\mathrm{pN}}\left(t_{\mathrm{peak}}\right)\;\right]\;$$

where *A*_Tot.uncorr_(*t*_peak_) is the peak radioactive concentration (kBq.mL^−1^), which is *not corrected for*^*18*^*F physical decay.*

### Z-F model

Zanotti-Fregonara et al. assessed ^18^F-FDG integrated activity in embryo assuming (a) instantaneous tracer uptake, (b) irreversible trapping and (c) the maximal SUV (hottest pixel; *corrected for*^*18*^*F physical decay*) recorded 60 min after the injection, could be taken as an initial activity concentration that exponentially decays with time [7,8]. In other words, the total number of disintegrations *Ã*_Z_ (no unit) occurring in embryo volume *V* (mL), was obtained from the area under the curve (AUC) of the function ‘*A*_Tot_(*t* = 60) * exp(−λt)’, which is

(10) $${\tilde{A}}_{Z}≅\;V*A_{\mathrm{Tot}}\left(60\right)/\lambda \;={\mathrm{SUV}}_{\mathrm{corr}}\left(60\right)*\;\;\left[\;V*\mathrm{ID}/\lambda *W\;\right]$$

Assuming irreversible trapping in our model, comparison with Z-F model, i.e. comparison of Equations 8 and 10, is equivalent to comparing two ratios, i.e. [AUC_NIF_ / λ*C*_pN_(*t*_peak_)] versus [exp(60λ) / λ], respectively.

Note that the two models lead to a very close final equation when it is assumed that (a) tracer is trapped irreversibly and (b) tracer plasma decay is tracer physical decay, as shown in the Appendix.

## Results

**Figure 1:**
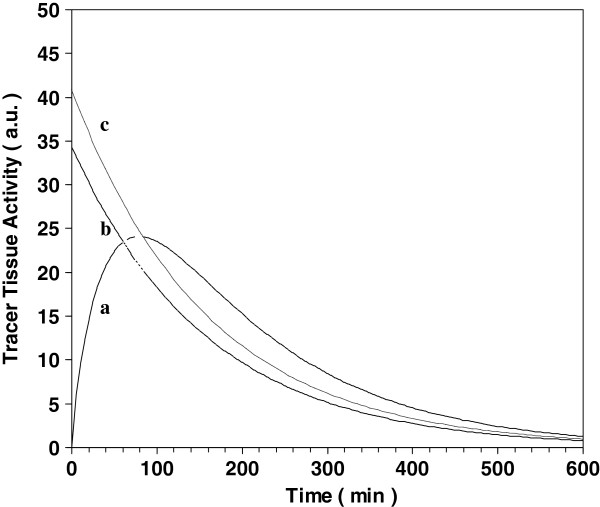
**Model comparison.** Curve (**a**) Trapped tracer activity (in arbitrary unit) versus time (in minutes) from Equation 3, assuming irreversible trapping, and the input function of Vriens et al. for ^18^F-FDG was used [15]. Curve (**b**) (full line) Z-F function, i.e. A_Tot_(*t* = 60) * exp(−λ*t*) (the value for A_Tot_(*t*=60) was taken from curve a). Curve (**c**) (dotted line) Z-F function with A_Tot_(*t* = 84) (instead of A_Tot_(*t* = 60)) that gives similar AUC for the two models.

Curve a in Figure 1 was obtained from Equation 3 for irreversible trapping, showing that tissue activity peaks at *t* = 79 min (leading to a [AUC_NIF_ / λC_pN_(t_peak_)] ratio of 269 min), by using median values for the time constants of the normalized ^18^F-FDG input function of Vriens et al. [15] [in comparison with Vriens et al. results (Table two-first column) the decay constants were modified in our model to take ^18^F physical decay into account]. Using minimal and maximal inter quartile values for these time constants leads to a [AUC_NIF_/λC_pN_(t_peak_)] ratio of 272 and 262 min, for tissue activity peak at 82 and 76 min, respectively. In other words, the relative difference between the ratio obtained and minimal and maximal inter-quartile values is 3.8%. An estimate of integrated activity occurring in a tissue volume (*V*, in mL) can be computed from either Equation 8 or 9, and using AUC_NIF_ / λC_pN_(t_peak_) = 269 min, as

(11) $${\tilde{A}}_{C}≅162\;{*10}^{5}\;*\;{\mathrm{SUV}}_{\mathrm{uncorr}}\left(t_{\mathrm{peak}}\right)*V*\mathrm{ID}/W\;$$

(12) $${\tilde{A}}_{C}≅162\;{*\;10}^{5}\;*\;A_{\mathrm{Tot.uncorr}}\left(t_{\mathrm{peak}}\right)*\;V\;$$

Curve a in Figure 1 also shows that when SUV_uncorr_ in Equation 11 (or peak radioactive concentration A_Tot.uncorr_ in Equation 12) is assessed at *t* = 55 min or *t* = 110 min after injection, it is 5% lower than that obtained at peak time *t* = 79 min.

Furthermore, Figure 1 compares the plots of the ^18^F-FDG TAC from Equation 3 (curve a) and of the function A_Tot_(*t* = 60) * exp(−λ*t*) from Z-F model (curve b). Curve b was plotted using the value of A_Tot_(*t* = 60) obtained in curve a. The area under each curve is the integrated activity assessed from each model, respectively. Figure 1 visually suggests that the two AUCs are very close. This visual interpretation is confirmed by the comparison between the ratios [AUC_NIF_ / λ*C*_pN_(*t*_peak_)] and [exp(60λ) / λ], which only differ by 16%: 269 min versus 232 min, respectively. In this connection, the total number of disintegrations occurring in the foetus obtained from the present study and from the Z-F model [8] (SUV_corr_(*t* = 60) = 4.5 g.mL^-1^, *V* = 21 mL, and 71-kg mother) is estimated to 14,720,000 and 12,623,000/MBq injected to the mother, respectively. Comparison between the [AUC_NIF_ / λ*C*_pN_(*t*_peak_)] and [exp(60λ) / λ] ratios further indicates that the two AUCs in Figure 1 would be equal if the SUV was acquired at *t* = 84 min after injection (curve c) (alternatively, the two AUCs can also be equal when a low ^18^F-FDG release occurs in the tissue of interest, resulting in a lower AUC of the tissue TAC and in a shift of peak time to *t* = 77 min, instead of *t* = 79 min).

For healthy grey and white matter, which reversibly trap ^18^F-FDG, the [AUC_NIF_ / λ*C*_pN_(*t*)] ratio is 185 and 189 min, for peak activity at *t* = 61 and 63 min [9,10], respectively. For the healthy brain, integrated activity was calculated from Equation 8, assuming that brain was 50% gray and 50% white matter, with 805-mL volume each (as 2.3% of a 70-kg patient, with a brain density of 1) [10,16]. For SUV_uncorr_(*t*_peak_ = 60) = 4 g.mL^−1^ (= (5.3 + 2.7) / 2, on average [17]) and *ID* = 37 MBq, the total integrated activity for healthy brain compares with that calculated by Mejia et al.: 7.97 versus 6.57 (±1.51) MBq.h [11].

For the healthy lung, which irreversibly traps ^18^F-FDG, integrated activity was calculated from Equation 12 and experimental literature data [18]. Assuming that lung volume is 1,120 mL (as 1.6% of a 70-kg patient, with lung parenchyma density of 1) [16], for A_Tot.uncorr_(*t*_peak_) = 1.62 kBq.mL^−1^ on average (Table one in [18]), the total integrated activity for healthy lung compares with that calculated by Mejia: 0.93 versus 0.86 (±0.10) MBq.h for an administered activity (*ID*) of 37 MBq [11].

For the healthy liver, which reversibly traps ^18^F-FDG, the [AUC_NIF_ / λ*C*_pN_(*t*)] ratio is 164 min, for peak activity at about *t* = 55 min (Figure two in [19]). Total peak activity is 8.6 kBq/mL, and hence SUV_uncorr_(*t*_peak_) = 1.7 g.mL^-1^, a value identical to that obtained by Minamimoto et al. [17]. The total integrated activity was calculated from Equation 12, assuming that liver volume is 1,280 mL (as 2.0% of a 64-kg patient, with liver parenchyma density of 1) [16]. The total integrated activity for healthy liver compares with that calculated by Mejia: 3.47 versus 4.14 (±1.09) MBq.h, for an administered activity (*ID*) of 37 MBq [11].

## Discussion

### Analytical solution of integrated activity

This theoretical work showed that the calculation of an estimate of integrated activity in an arbitrary tissue using one SUV value, or using one radioactive concentration value (Equation 8 and 9, respectively), is possible. However, this estimation requires the following: (a) the use of a population-based input function [15], which is involved in the [AUC_NIF_ / λ*C*_pN_(*t*_peak_)] ratio (Equations 8 and 9) and (b) the use of SUV, which is uncorrected for ^18^F physical decay (either to the time of injection or to the beginning of acquisition) and is assessed about its peak value. The value of ^18^F-FDG release rate constant in the tissue of interest plays a role in the peak timing and hence in the value of *C*_pN_(*t*_peak_) (indeed, Equation 3 shows that the peak timing depends on the release rate constant, on the physical decay constant, and on the time constants of the ^18^F-FDG IF, whereas the uptake rate constant plays a role in the SUV_uncorr_(t_peak_) amplitude). If the ^18^F-FDG release rate constant from the tissue is unknown, and hence if the SUV_uncorr_ peak time is unknown, assuming that *k*_R_ is negligible (i.e. an irreversible trapping) leads to an overestimate. In current clinical practice, this overestimate is more acceptable than an underestimate and can be very quickly computed as *Ã*_C_ = 162 * 10^5^ * SUV_uncorr_(*t*_peak_) * *V* * *ID*/*W* (Equation 11).

The use of the semi-quantitative SUV index obtained from a single acquisition for integrated activity estimation requires assumptions presented above, resulting in different origins of measurement uncertainty. The measurement uncertainty that is related to the product ‘162 * 10^5^’, i.e*.* related to the [AUC_NIF_ / λ*C*_pN_(*t*_peak_)] ratio, was estimated to be ±3.8%, i.e. the relative difference between the [AUC_NIF_ / λ*C*_pN_(*t*_peak_)] ratio obtained by using minimal and maximal inter quartile values for the time constants of the normalized ^18^F-FDG IF of Vriens et al. [15]. Although this relative uncertainty is low, it is suggested that it could be still reduced by using, in each patient, a more specific IF adjusted with a single blood sample, instead of a population-based input function, as proposed by authors [14,20]: in other words, Equation 7 could be used instead of Equation 8. Note that such a method could be applied in particular in hyperglycaemic patients, while SUV_uncorr_(*t*_peak_) (even if it is lowered owing to a high blood glucose level) should be used as such, with appropriate measurement uncertainty discussed below. First, in current clinical practice, a strict time delay between injection and acquisition cannot be always fulfilled to obtain SUV_uncorr_ peak value. However, curve a in Figure 1 shows that trapped tracer radioactive concentration, and hence SUV_uncorr_, smoothly peaks at *t* = 79 min, and at *t* = 55 min or *t* = 110 min after injection, i.e. a typical acquisition time window, its value is 5% lower than that obtained at peak time. Second, SUV in itself involves a relative measurement uncertainty, which is the same for SUV either corrected or uncorrected for physical decay. In a recent study, de Langen et al. [21] showed that SUVmax repeatability, and hence SUVmax measurement uncertainty, was significantly greater than that of SUVmean, i.e. SUV averaged over several voxels. Therefore, the use of SUVmean appears relevant for dosimetry purpose. It can be obtained over a tissue volume, exhibiting either homogeneous or heterogeneous ^18^F-FDG uptake, as well as over a volume of interest within heterogeneous uptake (in this connection, the brain that is built of white and grey matter may be considered as an example of heterogeneous uptake). The SUVmean measurement uncertainty can be estimated from Figure two C in De Langen et al. study showing a minimal-maximal repeatability of 13% to 30% (with 95% confidence limit), leading then to a relative measurement uncertainty of 9.2% to 21.2% (=13/2^1/2^ to 30/2^1/2^). However, it should be noted that the use of the SUVmax value over a tissue volume, instead of SUVmean, may provide an overestimate (that is more acceptable than an underestimate), and the SUVmax measurement uncertainty can be obtained in the study of de Langen [21].

As a summary, for estimation of integrated activity from Equation 11: SUV_uncorr_ may be averaged over the tissue volume and should be assessed between 55 and 110 min after injection. The total uncertainty of the estimate ranges between 18% and 30% (as 18 = 3.8 + 5 + 9.2 and 30 = 3.8 + 5 + 21.2, i.e. simply summing the measurement uncertainties of different origins, respectively), depending on the tissue uptake: the higher the uptake, the lower the uncertainty.

### Comparison with Z-F model

To the very best of our knowledge, the only previously published analytical solution for estimating integrated activity from one SUV value was that of Zanotti-Fregonara et al. in the framework of foetal dosimetry. This is the reason why the present model was compared to that of Z-F [7,8]. The results of the two models, assuming irreversible trapping, were found in very good agreement with only a 16% difference. It is suggested that this difference is very likely overestimated. Indeed, the model comparison indicates that the estimates would be equal if the SUV was acquired at *t* = 84 min after injection (comparison of the AUC of curve a to curve c in Figure 1). A 16% difference was found with SUV obtained at 60 min after tracer injection, but this time delay is that of the start of the whole imaging procedure and not that of the particular step of PET imaging that involved the tissue of interest (embryo). Zanotti-Fregonara et al. indicated that imaging was obtained from the base of the skull to the mid-thigh level (7 table positions, 3-min per position). If so, taking also into account the time duration of the CT, the actual time delay between injection and acquisition was very likely longer than 60 min, and hence closer to 84 min. As a summary, it is suggested that the agreement between the two models mainly comes from (a) the common assumption that SUV is assessed about the SUV_uncorr_ peak and (b) that the ^18^F half-life somewhat dominates the decay of the trapped ^18^F-FDG TAC, as visually shown by Figure 1. Nevertheless, it is suggested that the main benefit of the proposed model over the Z-F model is that reversible trapping is also addressed.

Furthermore, in the framework of foetal dosimetry, it should be noted that both models can only provide a rough estimate of the integrated activity, because several unknown factors may influence the ^18^F-FDG uptake by the foetal tissues. In particular, foetal blood glucose level depends on that of the mother because glucose molecules can pass through the placental barrier, and therefore it is reasonable to assume a similar fate for glucose analogue molecules like ^18^F-FDG molecules. However, a main limitation of the integrated activity estimation from the two models is that the foetal ^18^F-FDG IF may be different from that of the mother. In addition, it should be noted that the foetal dosimetry should also involve the bladder as a source region, because it is close to the foetus and it is filled with urinary ^18^F-FDG [22].

### Comparison with Mejia’s results

Calculated integrated activity estimates were found in good agreement with Mejia’s results for healthy brain, lung and liver that show various degrees of tracer trapping reversibility and various fractions of free tracer in blood and interstitial volume. Healthy brain reversibly traps ^18^F-FDG and *F* is much lower than the ratio *K* / (λ + *k*_R_): 4.6% and 5.9% for grey and white matter, respectively [9]. Healthy lung irreversibly traps ^18^F-FDG and *F* is not negligible in comparison with the ratio *K* / (λ + *k*_R_): 63% at peak time [18]. Healthy liver reversibly traps ^18^F-FDG and *F* is not negligible in comparison with the ratio *K* / (λ + *k*_R_): 26% at peak time [19]. Furthermore, it should be noted that neglecting reversibility of the ^18^F-FDG uptake in healthy brain and liver leads to an overestimation of integrated activity that can be approached by comparing the [AUC_NIF_ / λ*C*_pN_(*t*_peak_)] ratio obtained at *t* = 62 and 55 min to that obtained at *t* = 79 min (=269/187 and 269/164), which differs by 44% and 64%, respectively: the greater the release rate constant, the greater the overestimation.

Furthermore, Mejia et al. assume a two-exponential decay for lung and liver TAC and a five-exponential decay for brain TAC that can be applied to experimental tissue data, respectively [11]. In comparison, the present study assumes a multi-exponential decay of the tracer IF leading to an analytical expression for integrated activity (Equation 4) that involves the sum ‘Σ(λC_i_/α_i_)’, which is the AUC of the tracer IF. This sum is close to the sum expressed in the right hand side of Equation A4 obtained by Mejia [11], thus suggesting that for integrated activity estimation, assuming a multi-exponential decay of the tissue TAC should be implicitly connected to assuming a multi-exponential decay of the tracer IF.

## Conclusions

This theoretical work showed that an estimate of cumulated activity in an arbitrary tissue can be computed from an equation that involves the tissue SUV, which is used without physical decay correction and is assessed about its peak (i.e. SUV_uncorr_(*t*_peak_)). Furthermore, if the ^18^F-FDG release rate constant from the tissue is unknown, in other words, if peak time of SUV_uncorr_ is unknown, this work shows that assuming an irreversible trapping leads to an overestimate. This overestimate is more acceptable than an underestimate and can be very quickly computed as *Ã*_C_ = 162.10^5^ * SUV_uncorr_ * *V* * *ID*/*W* (*V*, tissue volume, in mL; *ID*, injected dose, in MBq; *W*, patient’s weight, in kg), where SUV_uncorr_ is a mean over *V* and is assessed between 55 and 110 min after injection. However, this calculation requires assumptions leading to a relative measurement uncertainty for the estimate that ranges between 18% and 30% (the higher the uptake, the lower the uncertainty). It is suggested that estimating ^18^F-FDG integrated activity using one SUV value could be helpful in circumstances such as accidental exposure, or for epidemiologic purposes such as in patients having undergone several examinations.

## Appendix

When the present model is developed assuming that (a) tracer is trapped irreversibly, i.e. *k*_R_ = 0, and that (b) tracer plasma decay equals the tracer physical decay, Equation 5 becomes:

(13) $${\tilde{A}}_{K}≅\;\left[V*K\;*C_{p}\left(t=0\right)\right]\;/\lambda$$

where *C*_p_(*t* = 0) is the number of tracer molecules per plasma unit volume (in mL^−1^) at the time of injection. Furthermore, when *k*_R_ = 0, at the trapped tracer peak Equation 6 becomes:

(14) KCptpeak=λCTraptpeak

When tracer plasma decay equals tracer physical decay, i.e. *C*_p_(*t*_peak_) = *C*_p_(*t* = 0) * exp(−λ*t*_peak_), combining Equations 1, 13 and 14 provides a further expression for ^18^F-FDG integrated activity in a tissue as follows:

(15) $${\tilde{A}}_{K}≅\;{\mathrm{SUV}}_{\mathrm{corr}}\left(t_{\mathrm{peak}}\right)\;*\;\left[\;V*\mathrm{ID}/\lambda \;W\;\right]$$

where SUV_corr_(*t*_peak_) is SUV that is *corrected for*^*18*^*F physical decay* and that is assessed at the trapped tracer peak, which is *t* = 160 min, as shown in a previously published work [23]. Comparison of Equations 10 and 15 shows they are very close except that SUV_corr_ is not assessed at the same time delay after injection, i.e. 60 and 160 min, respectively.

## Competing interests

The authors declare that they have no competing interests.

## Authors' contributions

EL conceived the model analysis, participated in the study design and coordination, and in the manuscript writing. MB participated in the study design, in the model interpretation and in the manuscript writing. JB participated in the study design, in the model interpretation and in the manuscript writing. RM participated in the study design, in the model interpretation and in the manuscript writing. All authors read and approved the final manuscript.
